# The Herbal Formula Granule Prescription Mahuang Decoction Ameliorated Chronic Kidney Disease Which Was Associated with Restoration of Dysbiosis of Intestinal Microbiota in Rats

**DOI:** 10.1155/2021/4602612

**Published:** 2021-06-23

**Authors:** Yao Ming, Sijing Cheng, Wen Long, Hong-Lian Wang, Chuanlan Xu, Xiaoyu Liu, Qiong Zhang, Sha Zhao, Xia Zou, Junming Fan, Li Wang, Hongchun Shen

**Affiliations:** ^1^Department of Endocrinology, The Affiliated Traditional Chinese Medicine Hospital of Southwest Medical University, Luzhou, Sichuan, 646000, China; ^2^Department of Nephrology, The Central Hospital of Dazhou City, Dazhou, Sichuan 635000, China; ^3^Department of Nephrology, The Affiliated Traditional Chinese Medicine Hospital of Southwest Medical University, Luzhou, Sichuan 646000, China; ^4^Laboratory of Organ Fibrosis Prophylaxis and Treatment by Combine Traditional Chinese and Western Medicine, Research Center of Combine Traditional Chinese and Western Medicine, Affiliated Traditional Chinese Medicine Hospital of Southwest Medical University, Luzhou, Sichuan 646000, China; ^5^Faculty of Medicine, The Chinese University of Hong Kong, Hong Kong, SAR 999077, China; ^6^Department of Clinical Laboratory, The Affiliated Traditional Chinese Medicine Hospital of Southwest Medical University, Luzhou, Sichuan 646000, China; ^7^Chengdu Medical College, Chengdu, Sichuan 610041, China; ^8^College of Integrated Chinese and Western Medicine, Southwest Medical University, Luzhou, Sichuan 646000, China

## Abstract

Chronic kidney disease (CKD) has become a global health issue, and there is increasing evidence showing the beneficial roles of traditional Chinese medicine (TCM) in CKD treatment. Here, we studied the renoprotective role of Mahuang decoction, a famous TCM prescription, in a rat CKD model induced with the combination of doxorubicin and adenine. Our data showed that intragastric administration of Mahuang decoction inhibited the loss of bodyweight and attenuated proteinuria, serum creatinine, and blood urea nitrogen in CKD rats. Kidney histological analysis revealed decreased tubulointerstitial injury and fibrosis in CKD rats treated with Mahuang decoction accompanied with suppressed expression of TGF-*β*1 and phosphorylated NF-*κ*B/P65 (p-P65) as indicated by immunohistochemistry. ELISA analysis demonstrated reduced serum levels of proinflammatory cytokines TNF*α* and IL-6. Most importantly, intestinal microbiota analysis by 16s rRNA-seq showed that Mahuang decoction restored the impaired richness and diversity of intestinal microflora and recovered the disrupted microbial community through reducing the abundance of deleterious microbes and promoting the expansion of beneficial microbes in CKD rats. Collectively, our findings demonstrated that Mahuang decoction mitigated kidney functional and structural impairment in CKD rats which were associated with the restoration of dysbiosis of intestinal microbiota, implying its potential in clinical CKD treatment.

## 1. Introduction

Chronic kidney disease (CKD) is characterized by long-term impairment of renal function (reduced glomerular filtration rate or increased excretion of urinary albumin or both) and has become a global health issue. The incidence of CKD is estimated to be 8–16% worldwide [1]. In China, the national cross-sectional screen revealed a prevalence of about 10.8% [2]. Without proper medical intervention, CKD can progress into end-stage renal disease (ESRD) associated with lethal cardiovascular complications. Although multiple treatments have been applied which mainly focus on the control of blood pressure, lipid, glucose, immune reaction, and lifestyle, the outcome remains poor with high cost while ESRD occurs in many patients [1]. Therefore, there is an urgent demand to develop new drugs for effective CKD therapy.

Intestinal microbiota constitutes an important part of the human body and maintains a symbiotic ecosystem in healthy conditions. The dysbiosis of intestinal microbiota is associated with diverse disorders including CKD in both patient and animal models [3, 4]. In CKD, impaired renal function leads to increased blood urea. The diffusion of urea into the gut lumen reshapes the biochemical milieu which facilitates the propagation of urease- and uricase-containing bacteria, leading to the production of ammonia and ammonium hydroxide which damage the integrity of intestinal mucosa and enhance its permeability [3, 5]. This results in the elevated level of intestinal bacteria-originated uremic toxins in the circulation, which causes systemic inflammation as featured in CKD [5, 6]. Furthermore, the changed intestinal luminal milieu may also facilitate the expansion of proteolytic bacteria which produce uremic toxins (e.g., indoxyl sulfate and p-cresol) and suppress the growth of saccharolytic bacteria which can generate short-chain fatty acids (SCFAs), a group of metabolites beneficial to intestinal mucosal barrier integrity and enterocyte energy supply. Thus, this further promotes the disruption of intestinal integrity [3, 7]. Animal and clinical studies demonstrated that modification of intestinal microbiota through dietary intervention or supplement of prebiotics or probiotics can attenuate serum levels of uremic toxins, inflammation, and the severity of CKD [8, 9].

Traditional Chinese medicine (TCM), mainly in the form of herbal medicine, is widely used to treat diseases including CKD in China and other countries in east Asia. During the thousands of years' practice, many classical TCM prescriptions have been developed for the clinical treatment of kidney disease. Moreover, numerous preclinical studies and clinical trials have validated the renoprotective roles of specific TCM prescriptions, individual TCM herbal ingredients, or its derivatives [10–12]. Recently, a large-scale retrospective cohort study reported that TCM is beneficial for the long-term survival of CKD patients [11].

Mahuang decoction (“*Mahuang Soup*” in Chinese) is a famous prescription recorded in TCM document *Treatise on Febrile Diseases*. Mahuang decoction consists of four herbal ingredients, *Ephedrae Herba* (Mahuang), *Cinnamomi Ramulus*, *Armeniacae Semen Amarum*, and *Glycyrrhizae Radix et Rhizoma Praeparata cum Melle*. According to the TCM theory, Mahuang decoction functions by stimulating perspiration, enhancing the breath of the lung, and mitigating asthma. In clinical practice, Mahuang decoction is usually used to treat cold, chronic bronchitis, bronchial asthma, and so on [13]. In this study, we firstly investigated the renoprotective role of Mahuang decoction in CKD.

## 2. Methods

### 2.1. Drugs

The standard prescription of Mahuang decoction is composed of 28.6% of *Ephedrae Herba* (w/w), 34.2% *Cinnamomi Ramulus* (w/w), 28.6% *Armeniacae Semen Amarum* (w/w), and 8.6% *Glycyrrhizae Radix et Rhizoma Praeparata cum Melle* (w/w). All individual herbal drugs were purchased in the form of herbal formula granules manufactured by Sichuan Neo-Green Pharmaceutical Development Co., Ltd (China) in compliance with the standards of national good manufacturing practices (GMP, V.2010) released by the Health Ministry of China. Herbal formula granule was made from single conventional herbal medicine by water extraction, concentration, and drying, which has equivalent pharmaceutical effects compared with its original herbal medicine and is more convenient and stable. The individual herbal formula granules were blended in warm water freshly before animal administration.

### 2.2. CKD Rat Model and Drug Administration

12-week-old male Sprague Dawley (SD) rats were purchased from the Animal Experimental Center of Southwest Medical University and subject to adaptive feeding for 1 week. Rats were maintained in a specific-pathogen free facility with 12 h light/dark cycle and free access to food and water. For CKD model construction, the rats were given a single intravenous injection of 4 mg/kg doxorubicin (Zhejiang Hisun Pharmaceutical Inc., China) through tail vein followed by daily administration of 200 mg/kg adenine (Sigma, cat# A8626, USA) by gavage for 4 weeks. The rats with 24 h proteinuria larger than 50 mg were considered successful in CKD induction. Then, the CKD rats were randomly allocated into 5 groups: CKD model group (*n* = 10), Mahuang decoction treatment groups (low, middle, and high dose, *n* = 10, respectively), and valsartan treatment group (*n* = 10, positive control group). Rats in the Mahuang decoction treatment group were subject to the administration of 0.3 g/kg/day (low dose) or 0.6 g/kg/day (middle dose) or 1.2 g/kg/day (high dose) of Mahuang decoction by gavage for 4 weeks. Rats in the valsartan group were treated with 13 mg/kg/day valsartan by gavage for 4 weeks, while rats in the CKD model group were treated with saline in the same way. In addition, a group of healthy rats (*n* = 10) with the treatment of the only saline was used as normal control.

At the end of the treatment, 24 h urine was collected in a metabolic cage for each rat. After anesthesia with pentobarbital sodium (50 mg/kg), the rats were sacrificed by cervical dislocation. Blood was drained through the abdominal aorta followed by serum isolation by centrifugation and stored at −80°C. The kidneyswere fixed in 4% paraformaldehyde for 24h followed by paraffin embedding. All animal experiments in this study were approved and carried out in accordance with corresponding regulations of the animal experimental ethics committee of the Southwest Medical University.

### 2.3. 24 h Proteinuria, Serum Creatinine, and BUN

Quantification of 24 h proteinuria, serum creatinine, and blood urea nitrogen (BUN) was performed by an automatic biochemical analyzer (Mindray, BS-380, China).

### 2.4. HE and Masson's Trichrome Staining

HE and Masson's trichrome staining were performed with the HE staining kit (Beyotime, cat# C0105, China) and Masson staining kit (Nanjing Jiancheng Biotech. cat# D026, China) according to the manufacturer's instruction, respectively. The renal tubulointerstitial injury index was calculated by considering the degree of tubular structure abnormality, tubular protein cast, interstitial inflammation, and fibrosis based on the HE and Masson's trichrome staining as previously described [14]. The fibrotic area was quantified with Image *J* software (NIH, USA).

### 2.5. Immunohistochemistry-IHC

The 4 *μ*m paraffin kidney sections were deparaffined in xylene and rehydrated in gradient ethanol. Endogenous peroxidase was blocked in 3% H_2_O_2_ for 30 min. Antigen retrieval was performed in 0.01 M citrate acid solution (pH 6.0) for 10 min in microwave. Next, the sections were blocked with 2.5% BSA for 1 h followed by primary antibody incubation overnight at 4°C. The next day, the sections were washed with PBS 3 times for 5 min each time. Then, the biotin-streptavidin detection system (ZSGB-Bio, cat# SP-9000, China) was used to probe the primary antibody following the recommended instruction. The signals were developed with 3,3′-diaminobenzidine (DAB) with the nucleus counter-stained with hematoxylin. Photos were captured with a light microscope equipped with a camera (Leica, DM500, Germany). Image *J* 1.47v software was used to quantify the staining intensity or positively stained nucleus. Primary antibodies used were anti-TGF-*β*1 (Boster-Bio, cat# BA0290, China, dilution ratio 1 : 100) and anti-phosphorylated NF-*κ*B/P65 (Santa Cruz, cat# sc-135769, USA, dilution ratio 1 : 100).

### 2.6. ELISA

Serum TNF*α* and IL-6 levels were determined with rat TNF*α* ELISA kit (Newbioscience, cat# ERC102a, China) and IL-6 ELISA kit (Newbioscience, cat# ECR003, China) according to the manufacturer's instruction.

### 2.7. 16S rRNA Gene Sequencing

For microbiome analysis, fresh feces were collected during the grabbing operation before sacrificing the animals and stored at −80°C. Total fecal DNA was isolated with the fecal genomic DNA isolation kit (Tiangen Biotech. cat# DP382, China). The V3 region of the 16S rRNA was amplified. The amplicon library was constructed with the Ion Plus Fragment Library Kit (Thermo Scientific, cat# 4471252, USA) followed by sequencing with Ion PGM™ System (Thermo Scientific, cat# 4462921, USA). Sequencing data was filtered with FASTX Toolkit 0.0.13 software, and clean reads were aligned with the Silva database by QIIME software to generate operational taxonomic units (OTUs). The microbiota diversity analysis (Shannon diversity index, rarefaction curve) was performed by Mothur software (release 1.36.0). To study the microbiota difference among different samples, a weighted UniFrac distance matrix was generated with Mothur software. Then, the weighted UniFrac PCoA was conducted with QIIME software. Permutation multivariate analysis (ERMANOVA) with Adonis test was performed based on weighted UniFrac distance matrix in RStudio software. Population constitution analysis of representative samples was generated by directly plotting the bacterial proportion at the family level by GraphPad Prism software.

### 2.8. Statistical Analysis

GraphPad Prism software was used for graph plotting and statistical analysis unless where indicated. Data were presented as the means ± SD. One-way ANOVA was used for multiple comparisons of quantitative data.

## 3. Results

### 3.1. Mahuang Decoction Improved Renal Function in CKD Rats

**Figure 1:**
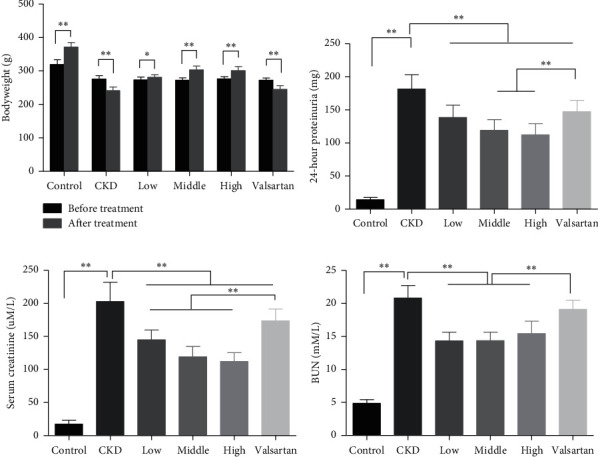
Mahuang decoction suppressed the bodyweight loss and ameliorated the impaired kidney function in CKD rats. (a) Bodyweight change of rats before CKD induction and after treatment. 24-hour proteinuria (b), serum creatinine (c), and blood urea nitrogen (BUN) (d) were quantified at the end of the experiment in the six groups of rats. ^*∗*^, *p* < 0.05. ^*∗∗*^, *p* < 0.01. In (a), *n* = 8 for the CKD group after treatment and *n* = 10 for the other groups. In (b)–(d), *n* = 8 for the CKD group and *n* = 10 for the other groups.

To explore whether Mahuang decoction would protect renal function in CKD, we firstly induced CKD in rats with a combination of doxorubicin and adenine as described in the method. The CKD rats were then given Mahuang decoction by gavage at a dose of 0.3 (low), or 0.6 (middle), or 1.2 g/kg/day (high) for 4 weeks. A group of CKD rats receiving valsartan (13 mg/kg/day), an angiotensin II receptor antagonist showing renoprotective function in CKD [15], was included to serve as a positive control. Each group contained 10 animals. Two animals died of severe end-stage renal disease in the CKD group without treatment. As shown in Figure 1(a), the CKD rats showed remarkable bodyweight loss at the end of the experiment compared with the start time point. However, Mahuang decoction treatment not only prevented the bodyweight loss but also slightly increased the bodyweight at all dose levels in CKD rats, indicating improved life quality. Nevertheless, valsartan failed to prevent bodyweight loss in CKD rats.

At the end of the treatment, CKD rats had significantly impaired renal function as demonstrated by remarkably increased proteinuria, serum creatinine, and blood urea nitrogen (BUN) compared with the control healthy rats. Mahuang decoction treatment notably reduced the proteinuria and serum creatinine in a dose-dependent manner in CKD rats. BUN level also decreased upon Mahuang decoction treatment although no dose-dependent effect was observed (Figures 1(b)–1(d)). Moreover, Mahuang decoction revealed a relatively better renoprotective effect than valsartan (Figures 1(b)–1(d)).

### 3.2. Mahuang Decoction Ameliorated Structural Injury of the Kidney in CKD Rats

**Figure 2:**
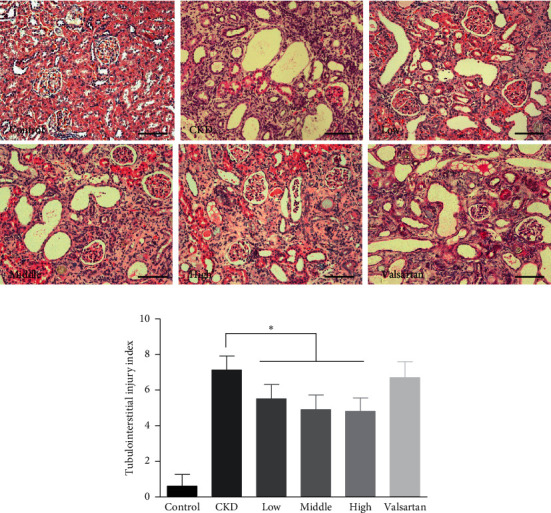
HE staining to show the tubulointerstitial injury in the kidney. (a) Representative HE images of the kidney were shown for the six groups of rats. Scale bar, 100 *μ*m. (b) Quantification analysis of the tubulointerstitial injury index. ^*∗*^, *p* < 0.05. *n* = 8 for the CKD group and *n* = 10 for the left groups.

Next, we investigated whether the improvement of renal function was accompanied by amelioration of structural injury of the kidney tissue in CKD rats treated with Mahuang decoction. As shown in Figure 2(a), HE staining of CKD kidney section revealed robust dilation of tubules and expansion of the interstitial area. On the contrary, treatment with Mahuang decoction mitigated the dilation of tubules (especially in the high dose group) and decreased the expansion of the interstitium. However, valsartan had weak benefits on the improvement of kidney structure in CKD. Semiquantitative analysis based on the severity of tubulointerstitial injury also showed that the overall structural abnormality was ameliorated after Mahuang decoction treatment although without dose-dependent effect (Figure 2(b)).

### 3.3. Mahuang Decoction Treatment Suppressed Fibrosis and Inflammation in CKD Rats

**Figure 3:**
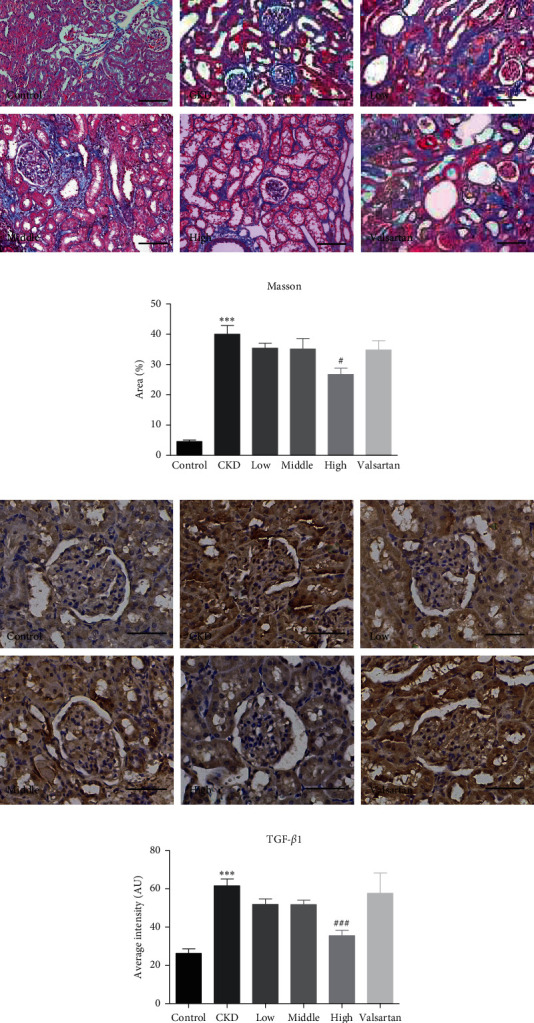
Mahuang decoction treatment mitigated the fibrosis of the CKD kidney. (a) Representative images of Masson's trichrome staining were shown for the six groups of rats. Scale bar, 100 *μ*m. (b) Quantification analysis of the fibrotic area. *n* = 8 for the CKD group and *n* = 10 for the left groups. (c) Representative images of IHC staining TGF-*β*1 in the kidney sections. Scale bar, 50 *μ*m. (d) Quantification of the staining intensity of TGF-*β*1 in (c). *n* = 3 animals for each group. In (b) and (d), ^*∗∗∗*^*p* ≤ 0.001versus the control group and ^#^*p* < 0.05and ^###^*p* ≤ 0.001versus the CKD group.

Chronic fibrosis and inflammation are key drivers of CKD progression [16, 17]. We next explored whether Mahuang decoction could improve fibrosis and inflammation in CKD rats. Masson's trichrome staining was used to evaluate the fibrosis of kidney tissue. As shown in Figure 3(a), CKD rats showed massive fibrosis in the peritubular region. After treatment with Mahuang decoction, the fibrosis was mitigated, which was more pronounced in the high dose group. Quantification of the fibrotic area also confirmed the relieved renal fibrosis in the Mahuang decoction (high dose)-treated rats (Figure 3(b)). Furthermore, IHC analysis indicated massive expression of TGF-*β*1 in the CKD kidney which was dose-dependently suppressed by Mahuang decoction treatment, while valsartan treatment has a weak influence on TGF-*β*1 expression (Figures 3(c) and 3(d)).

**Figure 4:**
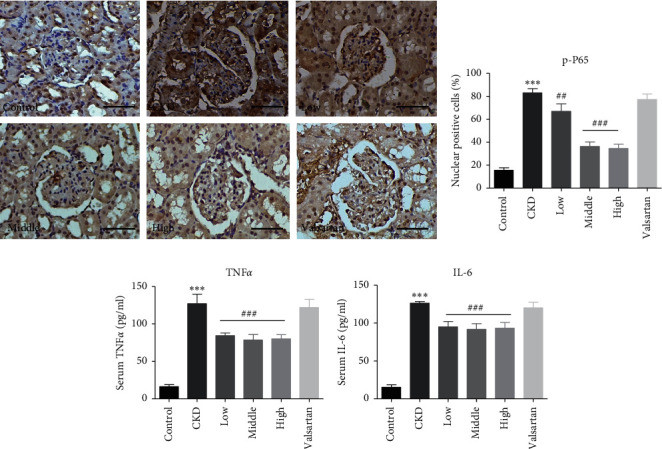
Mahuang decoction attenuated the inflammation in CKD rats. (a) IHC to show the expression of p-P65 in the kidney sections. Scale bar, 50 *μ*m. (b) Quantification of the percentage of P65 nuclear-positive cells in (a). *n* = 3 animals for each group. Serum levels of TNF*α* (c) and IL-6 (d) were determined by ELISA. In (c) and (d), *n* = 8 for the CKD group and *n* = 10 for the left groups. In (a)–(d), ^*∗∗∗*^*p* ≤ 0.001versus the control group and ^##^*p* < 0.01and ^###^*p* ≤ 0.001versus the CKD group.

Using IHC, we also detected the expression of phosphorylated NF-*κ*B/*p*65 (p-P65), the master regulator of the NF-*κ*B inflammatory pathway [18]. As illustrated in Figures 4(a) and 4(b), CKD rats showed significantly increased cells with nuclear staining of p-P65, which was mitigated by Mahuang decoction suggesting the inhibition of inflammatory pathway. We further determined the levels of inflammatory cytokines in the circulation and found that CKD rats had remarkably elevated serum levels of TNF*α* and IL-6, which were partially reversed by Mahuang decoction treatment (Figures 4(c) and 4(d)). In contrast, valsartan showed minimal improvement of p-P65 expression and serum levels of TNF*α* and IL-6. These data indicate that Mahuang decoction can ameliorate fibrosis and inflammation in CKD rats with better efficacy than valsartan.

### 3.4. Mahuang Decoction Treatment Restored Dysbiosis of Intestinal Microbiota in CKD Rats

**Figure 5:**
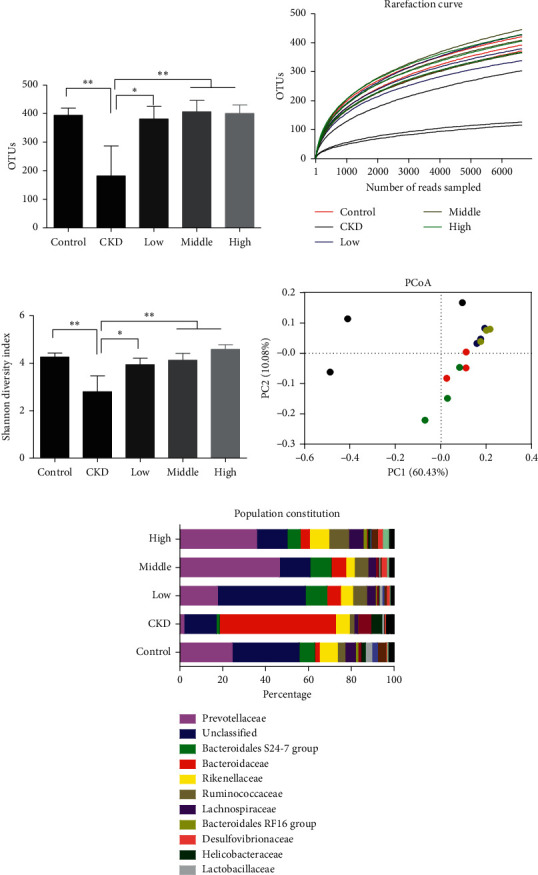
Mahuang decoction ameliorated intestinal dysbiosis in CKD rats. (a) Plotting of the total number of OUTs derived from 16s rRNA sequencing. (b) Rarefaction curve analysis of each sample. (c) Plotting of the Shannon index to analyze the diversity of microbiota. (d) Weighted UniFrac PCoA was used to visualize the population similarity. (e) Plotting of the bacterial proportion of representative samples at the family level. ^*∗*^*p* < 0.05. ^*∗∗*^*p* < 0.01. *n* = 3 for each treatment group in (a)–(d).

Intestinal microbial dysbiosis is reported to be associated with CKD, and restoration of the microbiota dysbiosis renders beneficial effects on renal function in CKD [8]. We next investigated whether intestinal microbiota was influenced in CKD rats treated with Mahuang decoction. We performed 16s rRNA sequencing with feces samples from healthy rats, control CKD rats, and CKD rats treated with Mahuang decoction, respectively. *In silico* analysis showed that, compared with the healthy rats, the richness of intestinal microbiota was largely reduced in CKD rats as revealed by decreased total identified OTUs in the feces sample (Figure 5(a)). The diminished microbiota richness was also evidenced by the rarefaction curve analysis (Figure 5(b)). Furthermore, CKD rats presented decreased taxon diversity of intestinal microbiota as indicated by Shannon index analysis (Figure 5(c)). However, the impaired bacterial richness and diversity were largely recovered in CKD rats treated with Mahuang decoction (Figures 5(a)–5(c)). In addition, the weighted UniFrac PCoA plotting revealed that CKD rats showed globally distinct intestinal microbial community structure compared with the healthy rats and Mahuang decoction-treated rats, although the statistical analysis showed no significance among groups (PERMANOVA with Adnois test, pseudo-F = 1.35, *R*^2^ = 0.35, *p* = 0.23) which may be caused by the small sample size (*n* = 3 for each group) and robust intragroup divergence especially in CKD rats (Figure 5(d)). Furthermore, plotting of the bacterial proportion at the family level in each group showed that CKD rats presented a notably disrupted intestinal bacterial constitution compared with that of the healthy rats. However, this disruption was generally mitigated in CKD rats treated with Mahuang decoction (Figure 5(e)).

### 3.5. Mahuang Decoction Treatment Promoted the Propagation of Beneficial Intestinal Bacteria but Suppressed the Expansion of Deleterious Bacteria

**Figure 6:**
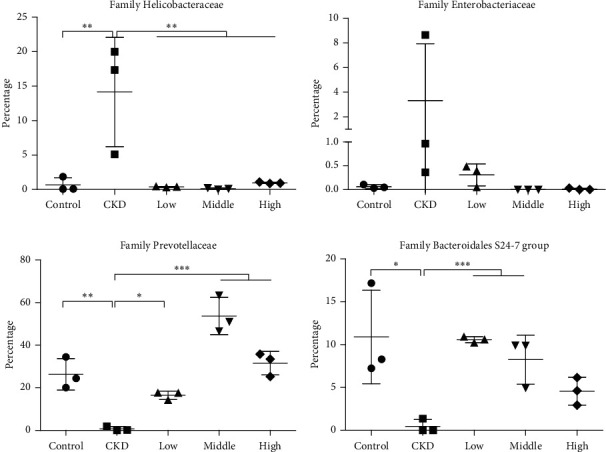
Plotting of relative abundance of the selected bacteria at the family level. *n* = 3 for each group. ^*∗*^*p* < 0.05. ^*∗∗*^*p* < 0.01. ^*∗∗∗*^*p* ≤ 0.001.

It is interesting to note that, among the bacteria with different richness between healthy and CKD rats at the family level, the abundance of Helicobacteraceae and Enterobacteriaceae was largely increased in the CKD rats (Figures 6(a) and 6(b)). It should be noted that bacteria from both of the two families possess the coding gene of urease and can convert urea into ammonia or ammonia hydroxide, which can damage the intestinal mucosa [3, 19]. Furthermore, bacteria from Enterobacteriaceae are responsible for the production of uremic toxins, like indoxyl sulfate and p-cresol sulfate [3]. Enterobacteriaceae bacteria also contribute to bloodstream infection in patients with hepatorenal failure [20]. On the other hand, the richness of a group of saccharolytic bacteria from Prevotellaceae and Bacteroidales S24-7, which can produce short-chain fatty acids (SCFAs) beneficial for intestinal epithelial integrity and function [3, 21], decreased remarkably in CKD rats (Figures 6(c) and 6(d)). In the healthy rats, Prevotellaceae and Bacteroidales S24-7 constituted an average of 26.35% and 10.9% of the microbiota, respectively (Figures 6(c) and 6(d)). However, their proportions decreased to less than 1% in CKD rats. In contrast, the disrupted abundance of the above bacteria was restored in CKD rats with the administration of Mahuang decoction. Collectively, CKD rats exhibited expansion of detrimental bacteria and contraction of probiotics while Mahuang decoction restored the disturbed balance of intestinal microbiota in CKD.

## 4. Discussion

The classical TCM prescription Mahuang decoction has been used for thousands of years to treat many diseases under the guidance of TCM theory in China. Up to now, no published study reported its role in kidney disease. In this study, we explored the therapeutic effects of Mahuang decoction in CKD rats induced by doxorubicin and adenine. Our data showed that Mahuang decoction ameliorated impaired function (reducing proteinuria, serum creatinine, and BUN) and structure (suppressing tissue injury and fibrosis) of the diseased kidney. Local (kidney) and systemic inflammations (blood) were also relieved. Most importantly, we found that Mahuang decoction restored the dysbiosis of intestinal microbiota, including recovery of the diversity and richness of intestinal flora, rebalance of the bacterial community by augmenting beneficial microbes, and suppressing detrimental microbes.

According to the principle of TCM theory, the therapeutic role of individual herbal medicine is ranked based on its contribution to disease treatment in a standard TCM prescription. In Mahuang decoction, *Ephedrae Herba* is believed to be the principal component. However, the role of *Ephedrae Herba* in kidney function remains controversial. It was reported that short-term infusion of ephedrine, one of the major pharmaceutically active extracts from *Ephedrae Herba*, promotes kidney function in humans [22]. Nevertheless, there is one case report showing that long-term consumption of *Ephedrae Herba* extract is associated with nephrolithiasis [23]. Combined with the findings in our study, it can be speculated that *Ephedrae Herba* indeed executes a renoprotective function, but the dose, medical formula, and delivery strategy should be carefully supervised in a disease-dependent context. The left three herbal medicines, *Cinnamomi Ramulus*, *Armeniacae Semen Amarum*, and *Glycyrrhizae Radix et Rhizoma Praeparata cum Melle* (also licorice), are believed to synergize the role of *Ephedrae Herba* in Mahuang decoction and are widely used herbal medicines in many other TCM prescriptions. Extracts of *Cinnamomi Ramulus* suppressed the activation of high glucose-stimulated mesangial cells and showed a renoprotective role in the experimental glomerulonephritis mouse [24, 25]. *Armeniacae Semen Amarum* was reported to protect the kidney from methotrexate-induced nephrotoxicity by eliminating oxidative stress [26]. Extracts of licorice protected the kidney from cisplatin-induced nephrotoxicity and sepsis-induced acute kidney injury [27, 28]. In consideration of the above multitherapeutic roles, we speculated that all the four herbal medicines in the Mahuang decoction contributed to the treatment of CKD in this study.

Temporary restrained inflammation and fibrosis contribute to the adaptive recovery of acute kidney injury. However, persistent and uncontrolled inflammation and fibrosis, which are common features of CKD, are harmful to long-term renal function [29]. Fibrosis is characterized by excessive deposition of extracellular matrix in the interstitial area leading to contraction and destruction of the functional renal structures. TGF-*β*/Smad signal dominates the profibrogenic mechanisms, where TGF-*β*1 is the principal causal profibrogenic ligand secreted by various cell types in the CKD kidney [17]. The CKD rats receiving Mahuang decoction demonstrated decreased fibrosis with attenuated TGF-*β*1 expression in the kidney, indicating that Mahuang decoction has antifibrotic activity by targeting TGF-*β*/Smad signaling (Figure 4).

As another important feature of CKD, inflammation is inversely related to renal function [16]. NF-*κ*B protein complexes are the center integrator of inflammatory signals [18]. The attenuated expression of NF-*κ*B/P65 in the kidney tissue indicated that Mahuang decoction can suppress the inflammatory signaling in the CKD kidney. Furthermore, chronic inflammation is proven to be systemic but not limited to the kidney. The proinflammatory cytokines and chemokines can come from extrarenal tissue, like fat, skeleton muscle, and liver [30, 31]. In recent years, intestinal bacteria-derived endotoxins are also recognized as an important source of proinflammatory factors in CKD [8, 16]. In this study, we observed increased propagation of urease- and endotoxin-producing bacteria Helicobacteraceae and Enterobacteriaceae and contraction of SCFAs-producing bacteria Prevotellaceae and Bacteroidales S24-7 (Figures 6(b) and 6(c)). This may enhance the permeability of intestinal epithelia and translocation of endotoxin (even pathogenic bacteria) into the circulation, resulting in augmented systemic inflammation [8,9]. The restored dysbiosis of intestinal microbiota and decreased circulatory levels of TNF*α* and IL-6 in CKD rats treated with Mahuang decoction implies that Mahuang decoction inhibits inflammation at least partially by suppressing the intestinal microbiota-derived proinflammatory insults (Figures 4(c)and 4(d)).

Among the bacteria with ectopic propagation, Helicobacteraceae attracts our interest as bacteria from this family are usually colonized in the stomach [8]. However, the relative richness of Helicobacteraceae increased averagely from 0.68% in healthy rats to 14.15% in CKD rats (Figure 6(a)). Bacterium from the Helicobacteraceae family is rarely reported to be associated with kidney disease except for*Helicobacter pylori*, which has a controversial relationship with CKD [32]. However, we failed to identify *Helicobacter pylori* at the species level in the fecal microbiota from CKD rats in our study (data not shown). This means that there exist unidentified bacterial species from the Helicobacteraceae family which may be associated with CKD. Enterobacteriaceae is another bacterial family showing increased richness in CKD rats in our study (Figure 6(b)). This is consistent with the clinical finding that Enterobacteriaceae presents an expansion in the intestinal microbiota of ESRD patients [3, 33].

Prevotellaceae and Bacteroidales S24-7 belong to Bacteroidetes, which is one of the three bacterial phyla dominating human and animal intestinal microflora [21, 34, 35]. In our study, Prevotellaceae, whose richness was robustly diminished in CKD, is the most abundant bacterial family in the healthy rats (Figure 6(c)). In line with our data, the decreased richness of Prevotellaceae was observed in CKD rats induced by 5/6 nephrectomy and in ESRD patients [33, 35]. Bacteroidales S24-7 also constitutes a considerable portion of the microflora in healthy rats and presents significantly decreased richness in CKD rats (Figure 6(d)). In contrast to our data, it is reported that the richness of Bacteroidales S24-7 increases in ESRD patients and CKD rats [36, 37]. Given the evidence showing that Bacteroidales S24-7 may function as a prebiotic [21], the relationship between Bacteroidales S24-7 and CKD needs further investigation.

The TCM usually contains multiple active pharmaceutical components in a given prescription or a single herbal medicine and may target different pathological processes simultaneously during disease treatment. Therefore, we should note that the observed renoprotective effects of Mahuang decoction should be ascribed to the multiple active components which may synergistically target the various aspects of CKD pathogenesis but not limited to the intestinal microbiota. More studies based on purified active pharmaceutical components are needed for a deeper insight into the renoprotective mechanism of Mahuang decoction in CKD treatment in the future.

## 5. Conclusion

In conclusion, our study demonstrates that the Mahuang decoction mitigates kidney damage in CKD. The renoprotective role of Mahuang decoction is associated with the amelioration of dysbiosis of intestinal microbiota. These findings indicated the therapeutic potential of Mahuang decoction for clinical CKD treatment.

## Data Availability

The list of OTUs identified in each rat feces sample by 16S rRNA sequencing is available in the figshare repository at https://doi.org/10.6084/m9.figshare.8947478.v2. Other data would be available from the corresponding author upon reasonable request.

## Abbreviations

CKD: Chronic kidney disease
TCM: Traditional Chinese medicine
BUN: Blood urea nitrogen
ELISA: Enzyme-linked immunosorbent assay
ESRD: End-stage renal disease
SCFAs: Short-chain fatty acids
IHC: Immunohistochemistry
OUT: Operational taxonomic unit.
